# *Acanthamoeba castellanii* Uncoupling Protein: A Complete Sequence, Activity, and Role in Response to Oxidative Stress

**DOI:** 10.3390/ijms241512501

**Published:** 2023-08-06

**Authors:** Nina Antos-Krzeminska, Anna Kicinska, Witold Nowak, Wieslawa Jarmuszkiewicz

**Affiliations:** 1 Department of Bioenergetics, Faculty of Biology, Adam Mickiewicz University, Uniwersytetu Poznanskiego 6, 61-614 Poznan, Poland; anna.kicinska@amu.edu.pl (A.K.); wieslawa.jarmuszkiewicz@amu.edu.pl (W.J.); 2 Molecular Biology Techniques Laboratory, Faculty of Biology, Adam Mickiewicz University, Uniwersytetu Poznanskiego 6, 61-614 Poznan, Poland; witold.nowak@amu.edu.pl

**Keywords:** *Acanthamoeba castellanii*, mitochondria, uncoupling protein, functional expression in yeast system, phylogenetic analysis, oxidative stress

## Abstract

Uncoupling proteins (UCPs) are mitochondrial inner membrane transporters that mediate free-fatty-acid-induced, purine-nucleotide-inhibited proton leak into the mitochondrial matrix, thereby uncoupling respiratory substrate oxidation from ATP synthesis. The aim of this study was to provide functional evidence that the putative *Acucp* gene of the free-living protozoan amoeba, *A. castellanii*, encodes the mitochondrial protein with uncoupling activity characteristic of UCPs and to investigate its role during oxidative stress. We report the sequencing and cloning of a complete *Acucp* coding sequence, its phylogenetic analysis, and the heterologous expression of AcUCP in the *S. cerevisiae* strain *InvSc1.* Measurements of mitochondrial respiratory activity and membrane potential indicate that the heterologous expression of AcUCP causes AcUCP-mediated uncoupling activity. In addition, in a model of oxidative stress with increased reactive oxygen species levels (superoxide dismutase 1 knockout yeasts), AcUCP expression strongly promotes cell survival and growth. The level of superoxide anion radicals is greatly reduced in the Δ*SOD1* strain expressing AcUCP. These results suggest that AcUCP targeted to yeast mitochondria causes uncoupling and may act as an antioxidant system. Phylogenetic analysis shows that the *A. castellanii* UCP diverges very early from other UCPs, but clearly locates within the UCP subfamily rather than among other mitochondrial anion carrier proteins.

## 1. Introduction

Uncoupling proteins (UCPs), the members of the mitochondrial anion carrier protein (MACP) family, contribute to mitochondrial energy dissipation, mediating free fatty acid (FFA)-lipid peroxidation product-induced, purine nucleotide (PN)-inhibited proton re-uptake into the mitochondrial matrix [1,2]. The first discovered UCP1, also called thermogenin, was found in brown adipose tissue of newborn humans and hibernating animals and was proven to be responsible for heat production and adaptive non-shivering thermogenesis [3,4]. However, the discovery of UCP homologues, i.e., UCP2-5, in non-thermogenic mammalian tissues [5,6,7,8] and UCPs in all systematic groups of eukaryotes, including amoeboid and parasite protists, non-fermentative yeast and filamentous fungi, plants as well as invertebrates (e.g., insects) and vertebrates (fish, reptiles, and birds) [9,10,11,12,13,14,15,16] has changed the perception of the role of these mitochondrial proteins in the living world. The widespread presence of UCPs in non-thermogenic tissues of animals and plants and in unicellular organisms implies that the functions of these proteins may not be limited to thermogenesis, which is characteristic of UCP1 in mammalian brown adipocytes. However, the physiological functions of UCP1 homologues, including UCPs of unicellular eukaryotes, are still under debate, and some researchers see the designation of these proteins as authentic uncoupling proteins as premature [17].

The UCP of the amoeba *Acanthamoeba castellanii* (AcUCP) is the first and best functionally characterized UCP of unicellular eukaryotes [1,18,19,20,21,22,23,24,25]. In the isolated mitochondria of this amoeboid protozoon, AcUCP has been shown to mediate FFA- and hydroxynonenal-activated, PN-inhibited proton leak that can divert energy from oxidative phosphorylation [20,21,22,23,24,25]. The inhibition by PNs is dependent on the membranous ubiquinone (Q) redox state [20,24,25,26,27]. It has been shown that ubiquinol (QH_2_) but not oxidized ubiquinone (Q) functions as a negative regulator of AcUCP inhibition by PNs [25]. Therefore, it has been proposed that the Q redox state could be a universal metabolic sensor that modulates the PN inhibition of inducible UCP activity [1,15], as described previously for rat skeletal muscle (UCP3 and UCP2), brown adipose tissue (UCP1), and potato tuber (plant UCP) mitochondria [26,27,28]. It has also been shown that the exposure of *A. castellanii* cell cultures to cold increases the activity and protein levels of AcUCP, indicating that UCP could be a cold-response protein in unicellular eukaryotes [19]. It has been suggested that the UCPs of the mitochondria of unicellular organisms may play an antioxidative role by decreasing reactive oxygen species (ROS) formation, preventing fluctuations in the ROS level throughout the growth cycle of this organism [29,30].

Besides *A. castellanii* AcUCP, functionally characterized UCPs of other unicellular organisms include fungal UCPs of *Candida albicans* [31], *Candida parapsilosis* [32], and *Yarrowia lypolitica* [33], as well as protozoan UCPs of *Plasmodium berghei* [34], *Plasmodium yoelli yoelii* [35], and *Dictyostelium discoideum* [36].

Although genes coding for UCPs have been identified in plants and animals, evidence for the presence of UCPs in fungi and protists is mainly functional and immunological. Little is known about the genes encoding for the UCPs and their direct protein products in unicellular organisms. The only exception is a UCP-like protein in yeast *Y. lypolitica* [33]. Using heterologous expression in *S. cerevisiae*, it has been shown that an oxaloacetate carrier gene of *Y. lipolytica* encodes protein, which also displays an uncoupling activity stimulated by FFAs and inhibited by PNs. Thus, although a large amount of functional information is available, especially from studies of AcUCP in isolated *A. castellanii* mitochondria, little is known about the genes of unicellular eukaryotes that encode the putative UCPs.

*A. castellanii* is a small non-photosynthesizing free-living soil and freshwater amoeba that has attracted attention as a model organism for the study of unicellular eukaryotic cell life. *A. castellanii* is an ecologically, medically (as an opportunistic pathogen), and evolutionarily important member of *Amoebozoa*, a major taxonomic group that diverged from the animal/fungal lineage after the split from plants [37]. Mitochondrial physiology of *A. castellanii* shows features that are common to both lineages, including a plant-type mitochondrial respiratory chain with additional dehydrogenases and an alternative oxidase [38,39]. The completion of the *A. castellanii* genome sequencing project [40] has made it possible to assign the previous functional discoveries for AcUCP to its putative coding sequence. The aim of this study was to determine the complete structure of the *Acucp* gene and to provide functional evidence that it encodes a mitochondrial protein with uncoupling activity characteristic of UCPs and to study its impact on constant oxidative stress. We report a sequencing and cloning of the complete *Acucp* coding sequence, as well as the functional analysis of AcUCP in mitochondria, using a heterologous yeast expression system. AcUCP expressed in *S. cerevisiae* cells is targeted to mitochondria where it introduces uncoupling activity. In the superoxide dismutase (*SOD1)* knockout strain expressing AcUCP, we observe a restoration of growth potential and a significant decrease in superoxide levels. We also discuss the molecular properties of the protein product of the coding sequence annotated here as *Acucp* and its evolutionary relations.

## 2. Results

### 2.1. Acucp Coding Sequence and Translated Protein Sequence Analysis

The *A. castellanii* uncoupling protein coding sequence deposited in NCBI GenBank, designated XM_004334150.1, was derived from the previously described genomic sequence (NW_004457290) [40]. However, the preliminary comparison of predicted uncoupling protein sequence coded by XM_004334150.1 with other UCP proteins suggested that the predicted gene model (gene ID: 14912673, ACA1_040210) was annotated with incorrect start and stop codons. The UCP protein coded by this incomplete sequence would be devoid of the first transmembrane domain characteristic for all known UCPs. Therefore, we used the 5′ and 3′ RACE technique to obtain the complete coding sequence.

**Figure 1:**
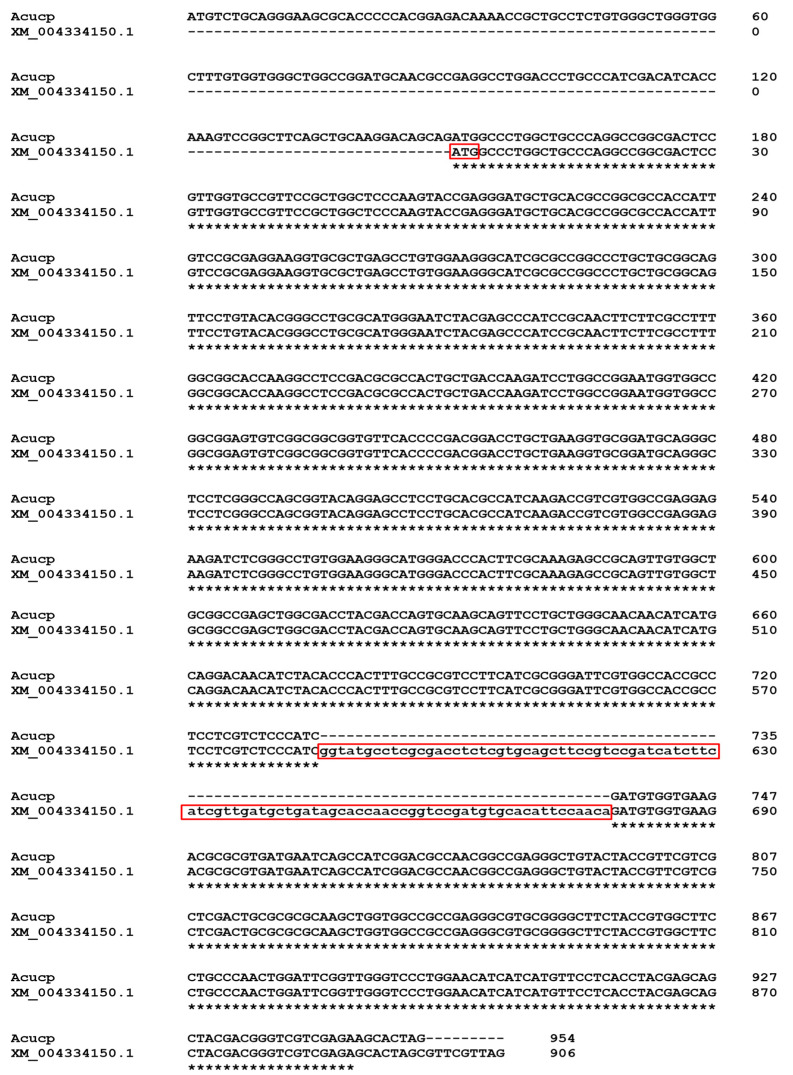
The multiple alignment of *Acucp* complete coding sequence and sequence annotated as XM_004334150.1. The figure presents (red frames) the misidentified start codon of the coding sequence and one misassembled intron (lower case) in XM_004334150.1 compared to *Acucp* complete coding sequence. Clustal Omega was used to generate the alignment. Asterisks indicate a complete match of the nucleotide sequences in the compared sequences.

After sequencing the RACE products, we obtained the sequence which was 150 nt longer at the 5′ end than previously available. The comparison of XM_004334150.1 and the complete sequence is shown in Figure 1. The start codon in XM_004334150.1 was probably misidentified by automatic bioinformatic tools, because in the upstream sequence there was an intron of a 79 nt length located between 136 and 214 nt. In addition, we found that the intron sequence in XM_004334150.1, covering a length of 93 nt between 586 and 679 nt, was misassembled in silico (Figure 1). The complete sequence was submitted to the NCBI GenBank and annotated as OR162004. 

**Figure 2:**
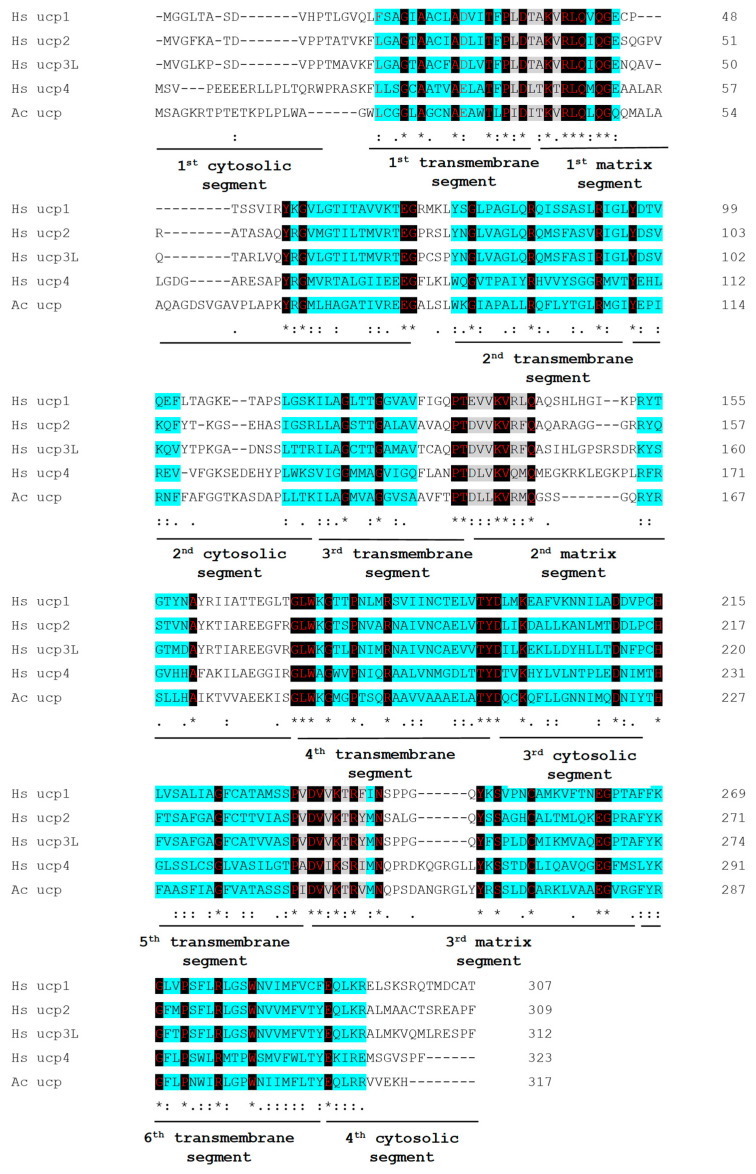
Multiple alignment of AcUCP in comparison to 4 isoforms of human uncoupling proteins. AcUCP, human UCP1 (NP_068605.1), UCP2 (AAC51336.1), UCP3 (AAC18822.1), UCP4 (NP_004268.3) sequences were used. The turquoise highlighted regions are highly conserved among the MACPs. The absolutely conserved residues (UCP signatures in all aligned sequences) are shown in red with black background. *—positions which have a single, fully conserved residue; “:”—conservation between groups of strongly similar properties; “.”—conservation between groups of weakly similar properties. Clustal Omega was used to generate the alignment.

By alignment of the genomic sequence (NW_004457290) with the obtained complete coding sequence (954 nt), we identified a total of five introns: 79 nt, first intron (13670–13748 nt); 68 nt, second intron (13869–13936 nt); 75 nt, third intron (14093–14167 nt); 87 nt, fourth intron (14351–14437 nt); 93 nt, fifth intron (14582–14674 nt) (Figure S1). After translation of the complete coding sequence, we obtained the AcUCP protein sequence consisting of 317 amino acids with characteristic features of both the uncoupling proteins and the MACP family, including a three-partite structure and six transmembrane domains. These features are presented in Figure 2, where we compared the obtained protein sequence with four human isoforms of uncoupling proteins. The search for conserved domains within the complete AcUCP protein sequence using BLASTp-NCBI-NIH revealed three MACP domains (pfam 00153 domains). The completely (or predominantly) conserved residues of the MACP family signatures were found in both transmembrane and matrix/cytosolic domains of AcUCP using Clustal Omega to generate the alignment (Figure 2). Completely conserved residues defined as UCP signatures [41] were found in all transmembrane domains, with special regard to the first and fourth transmembrane domains. The probability of location in the mitochondrion was determined as 90% and 100%, using the bioinformatic location prediction tools Yloc and MultiLoc, respectively (https://abi-services.cs.uni-tuebingen.de/yloc/webloc.cgi; https://abi-services.informatik.uni-tuebingen.de/multiloc2/webloc.cgi accessed on 20 September 2022). 

### 2.2. Detection of Expression of AcUCP in Yeast S. cerevisiae

The yeast *S. cerevisiae* model organism was chosen for the heterologous expression of AcUCP because yeast do not have their own UCP and therefore provides a good system for heterologous expression of UCPs from other organisms [9,42,43,44,45]. The complete AcUCP protein coding sequence (obtained at the 5′ and 3′ ends) with or without the N-His tag and the C-His tag was amplified by PCR using specific primers (see Materials and Methods and Supplementary Materials) and *A. castellanii* cDNA as a template. The sequences were afterwards cloned into the yeast-expressing vector pYES2 (pYES2 + *N-HisAcucp*, pYES2 + *C-HisAcucp*, pYES2 + *Acucp*) under a galactose-inducible promotor. *S. cerevisiae* cells (strain *InvSc1*) were transformed with the pYES2 + *Acucp* vector and its tagged versions (+AcUCP, AcUCP-expressing yeast) and with an empty pYES2 vector (-AcUCP, control AcUCP-deficient cells). In addition, Δ*SOD1* (BY4741 EUROSCARF) yeast cells were transformed with the pYES2 + *Acucp* vector and the empty pYES2 vector to test the effect of AcUCP expression on the yeast cell model under permanent oxidative stress.

**Figure 3:**
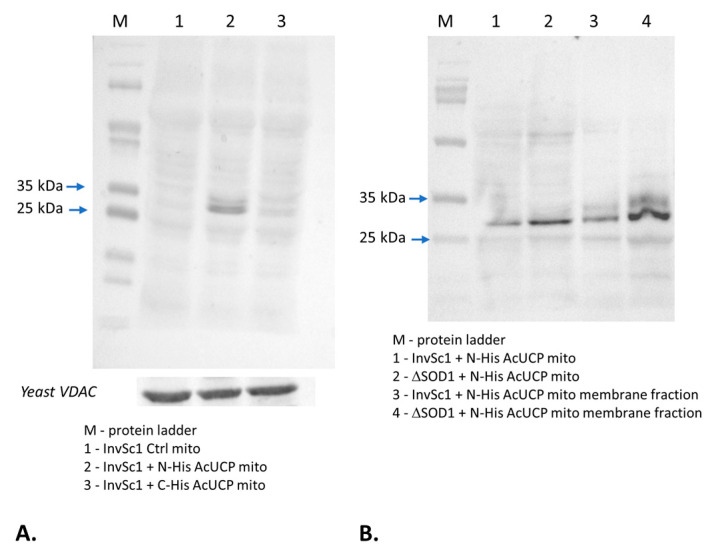
Immunodetection of AcUCP protein using Penta-His HRP Conjugate Kit antibody. (**A**) In *InvSc1* yeast mitochondria: 1—control (empty pYES2, Ctrl), 2—N-His tagged AcUCP-containing (pYES2 + N-His*Acucp*), 3—C-His tagged AcUCP-containing (pYES2 + C-His*Acucp*), 100 µg of mitochondrial proteins were loaded per lane, yeast anti-VDAC antibody was used as a loading control. (**B**) In *InvSc1* and Δ*SOD1* mitochondrial membrane fractions: 1—*InvSc1* N-His tagged AcUCP-containing mitochondria (pYES2 + N-His*Acucp*), 2—Δ*SOD1* N-His tagged AcUCP-containing mitochondria (pYES2 + N-His*Acucp*), 3—*InvSc1* N-His tagged AcUCP-containing (pYES2 + N-His*Acucp*) mitochondrial membrane fraction, 4—Δ*SOD1* N-His tagged AcUCP-containing (pYES2 + N-His*Acucp*) mitochondrial membrane fraction. Mitochondria were isolated from yeast cultured at 28 °C on the minimal selective medium without uracil supplemented with 3% glycerol and 2% D-galactose (inducing medium) for 18–20 h.

The presence of *Acucp* in transformed yeast cells was confirmed by PCR analysis and product sequencing. A specific 954 bp amplicon of *Acucp* was detected when *A. castellanii* cDNA, the pYES vector containing *Acucp*, and the extracts from *Acucp*-transformed yeast (+AcUCP yeast) were used as a template. To detect the AcUCP protein presence in the mitochondria of *Acucp*-transformed yeast cultured on glycerol and galactose, immunodetection was performed on isolated mitochondria using anti-His-tag antibody (Figure 3A). 

No AcUCP protein was found in control yeast mitochondria, while an ~32 kDa protein was detected in mitochondria from N-His tagged *Acucp*-transformed yeast (+ N-His-AcUCP mitochondria). In addition, we detected N-His tagged AcUCP in Δ*SOD1 +Acucp* mitochondria (Figure 3B). The protein’s presence in mitochondria treated with sodium carbonate suggests its membrane integration in both *InvSc1* and Δ*SOD1* strains expressing AcUCP. These results indicate that *Acucp* was successfully transformed to yeast and the AcUCP protein was targeted to yeast mitochondria. Because many reports show that tagged proteins may differ significantly from their wild-type counterparts in terms of activity and kinetic properties, we did not use a tagged version of the introduced *Acucp* coding sequence in other functional experiments [46,47,48,49].

### 2.3. Effect of AcUCP Expression on Yeast Growth

**Figure 4:**
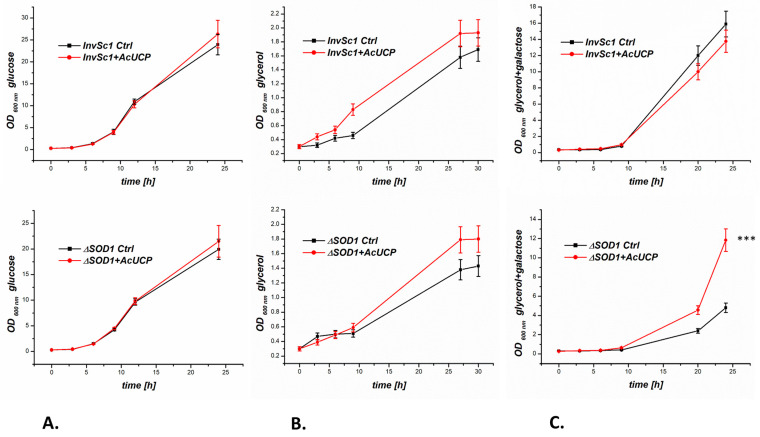
Growth curves of control (empty pYES2, Ctrl) and AcUCP-expressing (pYES2 + *Acucp*, +AcUCP) yeast *S. cerevisiae*, *InvSc1* and Δ*SOD1* strains. Yeasts were cultured at 28 °C on the minimal selective medium without uracil supplemented with (**A**) 2% glucose, (**B**) 3% glycerol or (**C**) 3% glycerol and 2% D-galactose (inducing medium). *** *p* < 0.001.

No significant changes were observed in the growth of the AcUCP-expressing and control (with empty pYES2) AcUCP-deficient *InvSc1* and Δ*SOD1* yeast strains when grown in a fermentable glucose medium (Figure 4A). Doubling times of the individual strains are listed in Supplementary Table S1. When grown in a non-fermentable glycerol medium (using mitochondrial metabolism), the *InvSc1* and Δ*SOD1* AcUCP-expressing strains showed a slight increase in growth (not significant) (Figure 4B). However, up to 18 h after induction of the AcUCP expression with galactose, AcUCP-expressing *InvSc1* yeast grew slower than control yeast (Figure 4C). In the case of the Δ*SOD1* AcUCP-expressing and control yeast strains, a large difference in growth rate was observed after induction of AcUCP expression with galactose. Since the Δ*SOD1* strain is under constant oxidative stress, it grows rather poorly on glycerol media. However, the Δ*SOD1* strain expressing AcUCP had a completely different growth pattern than the control strain 24 h after induction of expression, with a difference in growth rate of more than twofold in favour of the Δ*SOD1* yeast strain expressing AcUCP (OD_600_ = 11.84 ± 0.7 vs. 4.81 ± 0.27). Thus, the presence of AcUCP was extremely beneficial for the culture of *SOD1*-knockout yeast, possibly due to the reduction in the oxidative stress in the cells.

### 2.4. The Effect of Protonophoric Activity of AcUCP on Oxygen Uptake and Membrane Potential in Yeast Mitochondria

**Figure 5:**
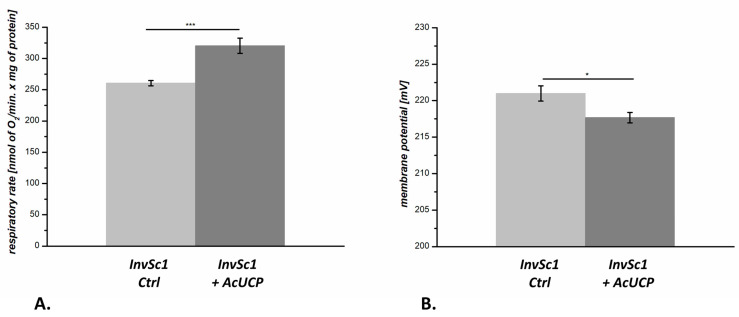
Comparison of oxygen consumption (**A**) and mΔΨ (**B**) of *InvSc1* control (empty pYES2, Ctrl) and AcUCP-containing mitochondria (pYES2 + *Acucp*, +AcUCP). Mitochondria were respiring with 40 µM NADH in self-regenerating system in state 4 (in the presence of oligomycin and carboxyatractyloside). * *p* < 0.05, *** *p* < 0.001.

The functional properties of mitochondria isolated from the control *InvSc1* (AcUCP-deficient) and AcUCP-expressing yeasts were examined to determine mitochondrial uncoupling activity. Comparison of mitochondrial respiratory activity and membrane potential (m∆Ψ) indicated that the AcUCP expression caused a slight uncoupling of the oxidative phosphorylation system in AcUCP-containing mitochondria. A slight decrease in the respiratory control ratio (RCR) was observed during oxidation of external NADH in mitochondria isolated from yeast expressing AcUCP (RCR = 2.05 ± 0.14*, SEM) in comparison to mitochondria from yeast transformed with the empty vector (RCR = 2.42 ± 0.18, SEM). In addition, under non-phosphorylating conditions (state 4 with 40 µM NADH and self-regenerating system in the presence of oligomycin and carboxyatractyloside (CATR)), we observed a marked ~20% increase in the respiratory rate for AcUCP-containing yeast mitochondria (320.2 ± 12.2 nmol O_2_/min/mg protein, SEM) compared to control yeast mitochondria (260.5 ± 4.4 nmol O_2_/min/mg protein, SEM) (Figure 5A). The increase in the respiratory rate was accompanied by a slight ~3 mV decrease in m∆Ψ in AcUCP-containing mitochondria (mΔΨ = 221.0 ± 1.0 mV, SEM, for control yeast mitochondria, and mΔΨ = 217.6 ± 0.7 mV, SEM, for AcUCP-containing yeast mitochondria) (Figure 5B). Under phosphorylating conditions (state 3), respiratory rate and m∆Ψ were similar in both types of mitochondria, indicating no change in phosphorylating respiration and mitochondrial respiratory chain capacity (Supplementary Table S2).

In *A. castellanii* mitochondria, the AcUCP activity is stimulated by FFAs (e.g., linoleic acid, LA) and inhibited by PNs, among which GTP has the strongest inhibitory effect [1,50]. Therefore, in the present study, the inhibition of mitochondrial proton conductance by GTP was considered diagnostic of the UCP function [50].

To exclude the uncoupling mediated by the ATP/ADP antiporter, all the non-phosphorylating respiration measurements were performed in the presence of CATR. We used 20 µM LA and 2 mM GTP to stimulate or inhibit AcUCP activity, respectively. However, no significant differences in the effects of the UCP modulators were observed when comparing control and AcUCP-containing mitochondria. In both types of mitochondria, linoleic acid similarly stimulated respiration and decreased m∆Ψ, while GTP inhibited respiration but did not restore mΔΨ (Supplementary Table S3).

Thus, the presence of AcUCP in yeast mitochondria resulted in a significant increase in respiratory rate and a decrease in mΔΨ under non-phosphorylating conditions, indicating that *Acucp* encodes mitochondrial UCP. However, regulation of AcUCP activity by FFA and PN could not be detected in the yeast model. 

### 2.5. AcUCP Expression Does Not Change Theoxaloacetate and Dicarboxylate Transport Activity in Yeast Mitochondria

**Figure 6:**
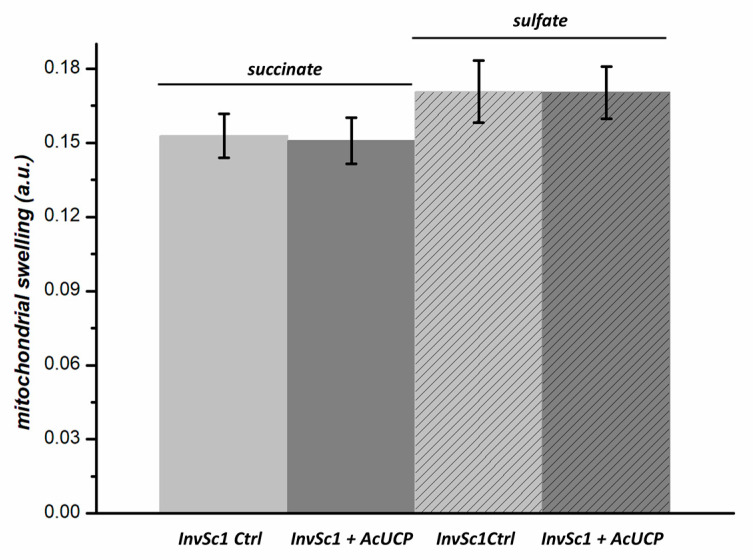
Transport activities of mitochondria isolated from *InvSc1* control (empty pYES2, Ctrl) and AcUCP-expressing (pYES2 + *Acucp*, +AcUCP) *S. cerevisiae*. The activities of the oxaloacetate carrier and the dicarboxylate carrier were determined from the swelling data. The rates of mitochondrial swelling were measured under conditions where swelling was directly dependent on oxaloacetate and sulphate transport (oxaloacetate carrier activity), as well as succinate transport (dicarboxylate carrier activity).

Since UCPs show sequence similarity to the other MACP family members, it has been previously suggested that other mitochondrial anion carriers may be responsible for the uncoupling activity in the mitochondria of unicellular organisms (e.g., fungus *Y. lypolityca*) [33] or that UCPs may have metabolite transport activity [51,52,53,54]. Mitochondrial swelling in iso-osmotic salts is used widely to determine the transport specificity of mitochondrial carriers, particularly those in yeast [55,56]. Swelling in ammonium sulphate can be used to measure the oxaloacetate transporter activity, while swelling in the ammonium succinate (plus phosphate) can be used to determine the dicarboxylate carrier activity. Therefore, we studied the transport of sulphate (oxaloacetate transporter activity) and succinate (dicarboxylate transporter activity) by measuring the swelling of control and AcUCP-containing yeast mitochondria. As shown in Figure 6, the transport activities of both transporters did not differ between control AcUCP-deficient and AcUCP-containing yeast mitochondria. These results demonstrate that the AcUCP expression product functions neither as a dicarboxylate transporter nor as an oxaloacetate transporter in yeast mitochondria.

### 2.6. The Influence of AcUCP Expression on Yeast Oxidative Stress Response

**Figure 7:**
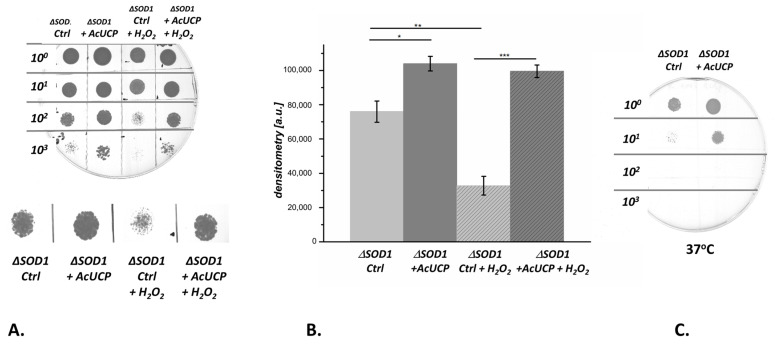
The effect of AcUCP expression on Δ*SOD1* yeast cell growth and survival under oxidative stress conditions. Growth of control (empty pYES2, Ctrl) and AcUCP-expressing (pYES2 + *Acucp*, +AcUCP) Δ*SOD1 S. cerevisiae* untreated or treated with 10 mM H_2_O_2_ for 2 h on MSM-URA, 3% glycerol, 2% galactose plates. (**A**) Growth of series of dilutions of yeast cultures. 10^2^ diluted colonies are shown enlarged. (**B**) Growth coverage within given spot of 100 times diluted (10^2^) cell cultures measured densitometrically. (**C**) Growth of control (empty pYES2, Ctrl) and AcUCP-expressing (pYES2 + *Acucp*, +AcUCP) Δ*SOD1 S. cerevisiae* cultured in temperature stress conditions (3 days at 37 °C). * *p* < 0.05, ** *p* < 0.01, *** *p* < 0.001.

AcUCP has a considerable impact on diminishing ROS production in *A. castellanii* mitochondria [29,30]. Therefore, we examined whether the presence of AcUCP affects the survival of yeast cells under oxidative stress conditions. Control and AcUCP-expressing Δ*SOD1* strains were treated with a final concentration of 10 mM H_2_O_2_ for 2 h. Cell growth and survival was monitored by following the ability of culture dilution series to grow on MSM-URA, 3%glycerol, 2% galactose selective plates (Figure 7A). The results were quantified by densitometric measurements of yeast growth coverage within a given spot (Figure 7B). In the absence of H_2_O_2_, the growth of AcUCP-expressing cells was approximately 40% better than that of control cells. After treatment with H_2_O_2_, AcUCP-expressing yeast cells grew three times faster than control cells. In addition, the growth of control Δ*SOD1* cells was severely arrested at 37 °C compared to Δ*SOD1* cells expressing AcUCP (Figure 7C). These results indicate that AcUCP targeted to yeast mitochondria may act as an antioxidative system under oxidative stress conditions. In the *S. cerevisiae* cells, which do not possess their own UCP and rely on other antioxidant systems to avoid ROS overproduction and oxidative stress damage, the effect of AcUCP expression is especially pronounced under severe oxidative stress in the absence of SOD1. As shown in Figure 4B, the Δ*SOD1* yeast strain transformed with the pYES + *Acucp* vector shows a considerable restoration of the growth potential compared to the impaired growth of the AcUCP-deficient strain with the Δ*SOD1* knockout. These findings show that AcUCP may complement yeast SOD1, an enzyme that catalyses the disproportionation of superoxide to hydrogen peroxide and dioxygen, likely by decreasing the overreduction of mitochondrial respiratory chain complexes and decreasing mitochondrial ROS formation.

### 2.7. Respiration of ΔSOD1 Control Cells and ΔSOD1 Cells Expressing AcUCP

**Figure 8:**
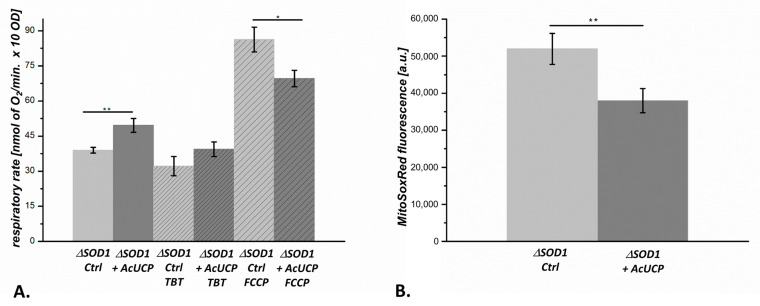
AcUCP expression effect on Δ*SOD1* yeast mitochondrial function. (**A**) The respiratory rate of control (empty pYES2, Ctrl)and AcUCP-expressing (pYES2 + *Acucp*, +AcUCP) Δ*SOD1 S. cerevisiae* cells. Measurements were conducted in SM-Ura medium with 3% glycerol and 3% galactose; TBT and FCCP were titrated to achieve maximal inhibition or stimulatory effect. (**B**) Determination of superoxide anion radical levels by MitoSoxRed fluorescence measurements in control (empty pYES2, Ctrl) and AcUCP-expressing (pYES2 + *Acucp*, +AcUCP) Δ*SOD1 S. cerevisiae* cells. * *p* < 0.05, ** *p* < 0.01.

The functional properties of control (AcUCP-deficient) and AcUCP-expressing Δ*SOD1* yeast cells were examined to determine mitochondrial uncoupling activity (Figure 8A). Δ*SOD1* yeast-expressing AcUCP showed increased basal respiration by approximately 27% compared to control Δ*SOD1* cells. Oxygen consumption rate in the presence of tributyltin (TBT), an ATP synthase inhibitor, increased slightly in AcUCP-expressing Δ*SOD1* cells, possibly indicating greater proton leakage, i.e., respiration unrelated to ATP synthesis. In addition, maximal oxygen consumption rate in the presence of *p*-trifluoro-methoxyphenylhydrazone carbonylcyanide (FCCP, an uncoupler) was reduced by approximately 24% in AcUCP-expressing Δ*SOD1* cells compared to control Δ*SOD1* cells, indicating a lower respiratory capacity of these cells. These results indicate that Δ*SOD1* + AcUCP yeast cells have greater mitochondrial uncoupling as a consequence of AcUCP expression.

### 2.8. Superoxide Anion Level in ΔSOD1 Cells Is Influenced by AcUCP Expression

Using the MitoSoxRed superoxide indicator, we analysed the level of superoxide in Δ*SOD1* control and Δ*SOD1* AcUCP-expressing yeast cells. The results showed an approximately 36% decrease in superoxide in Δ*SOD1* AcUCP-expressing cells, clearly indicating the antioxidant function of AcUCP in *SOD1* knockout yeast cells (Figure 8B).

### 2.9. AcUCP Phylogeny

It has been hypothesized that UCP4 is the ancestral UCP form, from which other UCPs diverged [41]. Other authors consider an early evolutionary divergence of UCPs (before the divergence of protostomes and deuterostomes) into three branches. These branches later gave rise to vertebrate and invertebrate UCP4 (first branch), vertebrate and invertebrate UCP5 (second branch), and invertebrate UCP6, together with vertebrate ancestral UCP that later diverged into vertebrate UCP1, 2, and 3 (third branch) [11]. 

**Figure 9:**
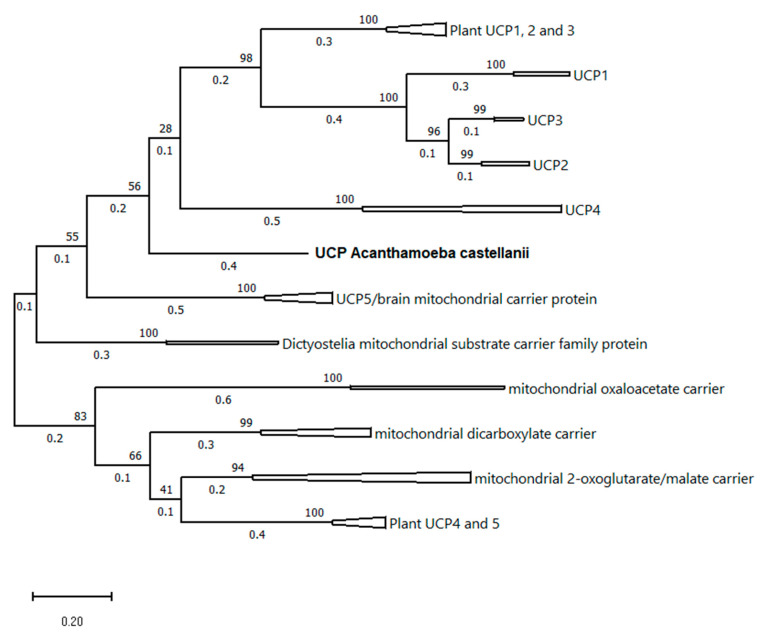
The phylogenetic relationships of MACPs with highest homology to UCPs. The multiple sequence alignments were conducted with the use of ClustalX. The results were obtained by Maximum Likelihood method and JTT matrix-based model. The tree with the highest log likelihood (−24,952.29) is shown. Evolutionary analyses were conducted in MEGA11. The complete maximum likelihood tree with bootstrap values is shown in Figure S2.

Knowledge of the AcUCP protein sequence has enabled construction of a phylogenetic tree, attempting evolutionary placement of the *A. castellanii* UCP protein sequence and comparing it with known uncoupling proteins’ sequences. The sequences of UCPs and some other MACPs have been carefully selected (based on the similarity to the AcUCP sequence—blastp search) to provide a possibly thorough and broad representation of different family members and taxonomic groups. A total of 85 amino acid sequences were used in the analysis, including *Amoebozoan,* plant, fungi, and animal (see Materials and Methods for accession numbers). As presented in Figure 9, the topology of the constructed maximum likelihood tree suggests that the AcUCP protein differs significantly from other UCPs, diverging very early. Our results show that there are two separated branches of UCPs, one including UCP4, plant UCP1, UCP2, and UCP3, and animal UCP1, UCP2, and UCP3, and a second including UCP5 proteins. It is worth noting that the *A. castellanii* protein diverged early, which is interesting because this protist possesses some plant cell characteristics (i.e., mitochondrial respiratory chain proteins—alternative oxidase [38] and alternative external/internal NAD(P)H dehydrogenases [39]). Other studied invertebrate UCP sequences are located in the UCP4 or the UCP5 branch. We show that the AcUCP protein is quite distant from other MACPs (with mitochondrial oxaloacetate and dicarboxylate carriers being the closest relatives). Also, the mitochondrial substrate carriers from other *Amoebozoans*, particularly *Dictyostelia*, are distantly related to the AcUCP protein and are located between UCP5 and other MACPs. The complete maximum likelihood tree with bootstrap values is shown in Supplementary Figure S2. 

## 3. Discussion

We used the *S. cerevisiae* expression system using the pYES2 vector as it has previously been successfully used for the expression of functional rat UCP1 and hummingbird UCP [9,27]. In our study, the pYES2 yeast expression allowed a modest, galactose-induced AcUCP expression. Stuart et al. suggested that the modest expression (using pYES 2) of UCP1 in yeast is a good experimental model for studying mammalian UCP1 function, in contrast to other overexpressing systems using stronger promoters, in which higher levels of expression can lead to artefactual uncoupling [57]. We confirmed the expression of AcUCP in yeast mitochondria by immunodetection using the antibody raised against His-tag. 

The function of AcUCP in isolated *A. castelanii* mitochondria has been carefully described [18,19,20,21,22,23,24,25,29,30]. The aim of this study was to link functional studies with a specific gene (*Acucp*) product by its expression in *S. cerevisiae*. In the present research, functional analysis indicates that AcUCP introduced to yeast mitochondria behaves as a protein with uncoupling activity. Because UCPs show a sequence similarity to the other MACP family members and some UCPs could have a metabolite transport activity [33,51,52,53,54], we studied the transport activities of AcUCP for sulphate (an oxaloacetate transporter activity) and succinate (a dicarboxylate transporter activity) in yeast AcUCP-containing mitochondria. Metabolite transport measurements indicate that the product of the *Acucp* expression does not work in yeast mitochondria as either a dicarboxylate carrier or an oxaloacetate carrier. Thus, the uncoupling activity is the only function observed in the mitochondria of transgenic yeast. The heterologous expression of AcUCP in *S. cerevisiae* allowed a direct characterization of the *Acucp* gene product and thus connected the previous functional data with a strictly defined protein function. This study provides the first evidence for the presence of putative UCP in unicellular organisms at the level of gene and its protein product. Evidence for the presence of UCPs in unicellular fungi, algae, and protists has so far been mainly functional and immunological. In sequenced genomes of the unicellular flagellate *C. reinhardtii* [58] and the amoeboid protist *D. discoideum* (DictyBase), genes encoding UCP(s) have been identified in silico, but no functional evidence has proven that they encode UCPs. Moreover, there are some discrepancies between phylogenetic data from high-throughput sequencing and functional studies. For example, in yeast *Y. lypolitica*, a UCP-like protein has been described, which is phylogenetically identified as an oxaloacetate carrier [33]. Using the heterologous expression in *S. cerevisiae*, it has been shown that an oxaloacetate carrier gene of *Y. lipolytica* encodes protein, which also displays an uncoupling activity stimulated by FFAs and inhibited by GDP [33]. It has been hypothesized that the *Y. lipolytica* oxaloacetate carrier may have evolved to allow FFA-induced uncoupling activity in contrast to the oxaloacetate carrier of *S. cerevisiae*. It remains unknown whether this putative dual function (combined oxaloacetate carrier and UCP activity) is found with an amino acid sequence resembling the oxaloacetate carrier structure in other UCPs of unicellular eukaryotes. Our study shows that this is not the case with UCP of *A. castellanii*. However, great care must be taken in concluding that the AcUCP protein has no other mitochondrial carrier function.

Although we did not observe FFA- and PN-specific regulation of AcUCP in transformed yeast mitochondria (in the presence of oligomycin and CATR), the same observation occurred in other UCPs derived from, for example, lamprey, amphioxus, skunk cabbage, or chicken and expressed in a yeast model [43,44,45,59]. It is important to mention that although UCP2 and UCP3 do have a regulated proton leak activity in animal tissues [60,61], they can hardly be activated or inhibited when moderately expressed in yeast mitochondria [62,63]. As a consequence, some research groups have suggested that this may be an artefactual uncoupling due to abnormal assembly in the yeast membrane, as previously described for mammalian UCP2 and UCP3 [57,62,63,64]. However, it should be noted that FFA can uncouple mitochondria independently of UCP activation, as other closely related mitochondrial anion carriers have been shown to be involved, albeit to a lesser extent, in the FFA cycle across the inner mitochondrial membrane. This has been demonstrated for the aspartate/glutamate [65] and dicarboxylate carriers [66]. On the other hand, the inhibition of AcUCP by GTP and the consequent recovery of mΔΨ may be hindered by high levels of ubiquinol (QH_2_), which act as a negative regulator of AcUCP inhibition by PNs [24,25].

Mild mitochondrial uncoupling prevents over-reduction in respiratory chain components and subsequent overproduction of ROS under conditions that favour reducing the power or phosphate the potential overflow [67,68,69,70,71,72]. It has previously been shown that FFA-induced uncoupling in the mitochondria of *A. castellanii* can prevent increased ROS generation by maintaining constant ROS levels throughout the growth cycle of this organism [29,30]. In the present study, the expression of AcUCP increased the survival of *SOD1* knockout yeast cells under oxidative stress conditions (mainly in the presence of H_2_O_2_). Furthermore, a significant restoration of growth potential was observed in the *SOD1* knockout yeast strain expressing AcUCP. Δ*SOD1* cells expressing AcUCP showed a relevant increase in basal respiration and proton leak-sustained respiration compared to the *SOD1* knockout control cells. Since superoxide anion can be used as a negative feedback loop for self-production by directly activating the proton leak through UCP1-3 [60], this may be the reason we observed a more pronounced mitochondrial uncoupling in Δ*SOD1* cells expressing AcUCP. Our results also showed a significant decrease in superoxide anion in Δ*SOD1* cells expressing AcUCP compared with control Δ*SOD1* cells. These results suggest that, as in the *A. castellanii* mitochondria, AcUCP may act as an antioxidant system when heterologously expressed in Δ*SOD1* yeast mitochondria. Therefore, similar to animal and plant UCP-mediated uncoupling [67,68,73], AcUCP-mediated uncoupling may prevent oxidative damage to cells. Our functional studies, indicating that AcUCP does not work in yeast mitochondria as a dicarboxylate carrier or an oxaloacetate carrier, are in good agreement with the presented phylogenetic analysis. Our results indicate that AcUCP is located in the phylogenetic tree between the two branches of UCP 1-4 and UCP5. The similarity to the other MACP family members is much lower and therefore the location of AcUCP is distant from that of other mitochondrial carriers. Our phylogenetic analysis, including unicellular UCP sequences, sheds new light on the evolution of UCPs. It suggests that UCP linage was split into at least two genetically distinct clades during a relatively early stage of evolution. The phylogenic position of AcUCP is supported by the phylogeny of the Eukarya domain which sets the Amoebozoa group as a basal branch within the Unikonts clade [74]. In summary, phylogeny analysis confirms the location of the *A. castellanii* protozoan UCP in the UCP subfamily. 

## 4. Materials and Methods

### 4.1. RACE 5′ and 3′ Ends of Acucp Coding Sequence Obtaining, Cloning in pYES2 Vector, Yeast Transformation

Total RNA from *A. castellanii* was isolated using the Trizol reagent (Sigma-Aldrich, St. Louis, MO, USA) with absorbance ratios of 260/280 and 260/230 above 2.0, and the first strand of cDNA was synthesised using RNA as template (GeneRacer™ Kit with SuperScript™ III RT and TOPO TA Cloning™ Kit for Sequencing ThermoFisher Scientific, Waltham, MA, USA). To capture the full-length 5′ and 3′ ends of the cDNA of the desired coding sequence, since the mRNA sequence, available in the GeneBank data-base (NCBI) and assigned as XM_004334150.1 was incomplete at both ends, the GeneRacer Advanced RACE kit was used. All gene-specific primers for XM_004334150.1 for 5′ RACE (GSPRev1: 3′AAGGCGAAGAAGTTGCGGAT5′ GSPRev2: 3′GTCAGCAGTGGCGCGTCGGA5′) and for 3′ RACE (GSPFd1: 3′ATGGCCCTGGCTGCCCAGGC5′ GSPFd2: 3′CAAGTACCGAGGGATGCTGC5′) were designed according to the core fragment sequence. Amplification and sequencing of RACE products allowed amplification and confirmation of the entire coding sequence (Supplementary Figure S3). The specific *Acucp* primers designed based on 5′ and 3′ RACE were used to amplify the coding sequence of *Acucp*. Primer sequences were as follows: *Acucp* fd: 5′ATGTCTGCAGGGAAGCGCA3′ and *Acucp* rev: 5′CTAGTGCTTCTCGACGACCC3′. After electrophoretic separation, the band of the desired size (954 bp) was excised from the gel and purified with the use of the Monarch^®^ PCR and DNA Cleanup Kit (New England BioLabs, Ipswich, MA, USA). The coding sequence of AcUCP was cloned to the TOPO TA Cloning^®^ Kit for sequencing, and its sequence was confirmed. Subsequently, re-cloning of the gene encoding UCP protein into the pYES2 vector (ThermoFisher Scientific, Waltham, MA, USA) was performed using the Vazyme ClonExpress II One Step Cloning system (Vazyme Biotech Co., Shanghai, China). The vector was linearized at the cloning site by its digestion with BamHI^HF^ and EcoRI^HF^ restriction enzymes (NEB). The inserts containing the coding sequence of the *Acucp* gene were prepared by way of one or two overhang PCRs. Primers with an additional “overhang” sequence at the 3′ ends were used to incorporate sequences homologous to the pYES2 vector and sequences coding six histidine residues with a glycine linker at the 5’ or the 3’ end of the inserts. The details about that recombination procedure are described in Supplementary Materials.

The *InvSc1* (MATa, his3Δ1, leu2, trp1-289, ura3-52, MAT, his3Δ1, leu2, trp1-289, ura3-52) or Δ*SOD1* (Y06913 from EUROSCARF, BY4741: MATa, ura3Δ0, leu2Δ0, his3Δ1, met15Δ0, YJR104c:kanMX4) strains of *S. cerevisiae* were transformed with empty pYES2 (control cells) or with construct pYES2+*Acucp* (+AcUCP cells) and their His-tagged versions by either electroporation or chemical transformation (Yeastmaker^TM^ Yeast Transformation System from Takara, San Jose, CA, USA). Selection was carried out on appropriate minimal selection medium plates (0.67% yeast nitrogen base, amino acids, and 2% glucose) without uracil (MSM—Ura). The presence of the *Acucp* gene in yeast cells was confirmed by PCR using specific *Acucp* starters and a yeast extract as a template.

### 4.2. Yeast Culture and Isolation of Mitochondria

Transformed *InvSc1* strains were selected by culturing on the minimal selective medium without uracil, inoculated from a single colony to 25 mL of liquid minimal selective medium containing 2% glucose and grown for 24 h at 28 °C under vigorous aeration (at 180 rpm agitation). Glucose repressed the *GAL1*-promoted gene expression. Afterwards, cultures were inoculated to 25 mL of medium containing 3% glycerol and cultured for 24 h to enable mitochondria generation. The culture was then inoculated into 850 mL of medium containing 3% glycerol and 2% D-galactose to induce the expression of *Acucp* gene. Both strains of yeast cells (control, AcUCP-deficient cells and +AcUCP, AcUCP-expressing cells) were harvested after 16–20 h of growth at the exponential phase of growth (OD_550_ ~2). For growth curve measurements, cultures were inoculated into a 25 mL medium containing 3% glycerol and 2% D-galactose. For mitochondria preparation, cells were centrifuged at 3000× *g* for 10 min and then washed in deionized water. Pellets were re-suspended in a buffer containing a 0.1 M Tris/Cl (pH 8.8) and a 10 mM dithiothreitol (DTT), incubated at 28 °C in an orbital shaker (125 rpm agitation) for 15 min, and washed in a 1.2 M sorbitol. The cells were re-suspended in 6 mL of a buffer containing a 1.2 M sorbitol, a 12 mM Tris/Cl (pH 7.4), and a 20 mM K/K phosphate buffer (pH 7.4) per 1 g of cells. Zymolyase was added (at 1 mg/g of wet weight of cells), and the suspension was incubated at 28 °C under a gentle agitation until approximately 90% of cells converted into spheroplasts (~30–50 min). The digestion was stopped by the addition of 1.2 M of ice-cold sorbitol and centrifugation. All subsequent steps were performed at 4 °C. Spheroplasts were pelleted, washed twice in the 1.2 M sorbitol, and then re-suspended in an isolation buffer (0.65 M mannitol, 20 mM Tris/Cl pH 7.4, 0.5 mM EDTA, 0.1 mM EGTA, 0.1% BSA, and 1 mM phenylmethylsulfonyl fluoride (PMSF)) at a ratio of 6 mL of buffer per 1 g of spheroplasts. After homogenization by ten passes with a tight Dounce homogenizer, homogenates were centrifuged at 1000× *g* for 10 min. The pellets were re-suspended, homogenized, and centrifuged again to collect the mitochondria remaining in the pellet. The supernatants were combined and centrifuged at 1000× *g* for 10 min. The resultant supernatants were centrifuged at 10,000× *g* for 10 min. The mitochondrial pellets were washed with a buffer containing 0.65 M of mannitol, 20 mM of Tris/Cl at pH 7.4, 0.2 mM of EGTA, and a 0.1% BSA, and then centrifuged at 10,000× *g* for 10 min. The final pellets were re-suspended in a small volume of the incubation medium (0.65 M of mannitol, 10 mM of HEPES, 10 mM of K/K phosphate buffer at pH 7.4, 5 mM of KCl, 2 mM of MgCl_2_, 0.5 mM of EGTA, and a 0.05% BSA). Mitochondrial protein concentrations were determined using the Bradford method.

### 4.3. AcUCP Immunological Detection

Mitochondria isolated from the control yeast containing an empty vector or N-His- and C-His-tagged AcUCP-expressing yeast were suspended in a sample buffer. For obtaining membrane fractions, 100 µg of each type of mitochondria were resuspended in 100 mM of sodium carbonate (pH 11.5) and centrifuged at 18,000× *g* for 10 min. The supernatant with the extracted soluble and peripheral membrane proteins was discarded. The pellet containing the purified mitochondrial membrane protein fractions was resuspended in a sample buffer. SDS-PAGE was performed using a 4.5 M urea 5% stacking gel and a 4.5 M urea 12% resolving gel with 100 µg of mitochondrial proteins loaded per lane. Separated mitochondrial proteins were then transferred onto the nitrocellulose membrane. Blots were blocked for 1 h with the Blocking Reagent in the Blocking Buffer (Qiagen, Hilden, Germany), according to manufacturer instructions probed with the Penta·His HRP Conjugate Kit (Qiagen, Hilden, Germany) and conjugated to horseradish peroxidase (HRP) at a dilution of 1:1000 for 1 h at room temperature. This kit is intended for sensitive detection of recombinant proteins carrying His tags, without the need for secondary antibodies. For VDAC immunodetection (used as a loading control), blots were blocked with 5% BSA in Tris-buffered saline overnight, probed with antibodies raised against yeast VDAC1 (a kind gift from Prof. Walter Neupert), and subsequently with goat anti-rabbit horseradish peroxidase-conjugated secondary antibodies (BioRad, Hercules, CA, USA) at dilutions of 1:1000 and 1:10,000, respectively, each for 1 h at room temperature. Protein bands were visualized using the Amersham ECL and G-box systems.

### 4.4. Mitochondrial Oxygen Consumption and Membrane Potential Measurements

For *InvSc1* mitochondria oxygen uptake was determined polarographically using a Rank Bros. (Cambridge, UK) oxygen electrode or a Hansatech oxygen electrode in either 2.8 mL or 1.4 mL of a standard incubation medium (at 28 °C), which consisted of 0.65 M of mannitol, 5 mM of KCl, 0.2 mM of MgCl_2_ (under non-phosphorylating conditions to increase the sensitivity of inhibition by GTP) or 2 mM of MgCl_2_ (under phosphorylating conditions), 0.5 mM of EGTA, 0.05% (*w*/*v*) BSA, 10 mM of HEPES, 10 mM of *K/K* phosphate buffer, at pH 6.9, with 0.4–1 mg of mitochondrial protein. O_2_ uptake values are presented in nmol O_2_ × min^−1^ × mg^−1^ protein. For Δ*SOD1* cell respiration measurements, the Hansatech oxygen electrode was used, and oxygen uptake was determined in 0.7 mL of selective medium: Ura with 3% glycerol and 2% galactose. O_2_ uptake values are presented in nmol O_2_ × min^−1^ × 10 OD_600_.

mΔΨ of the *InvSc1* mitochondria was measured simultaneously with the oxygen uptake (Rank Bros.) in 2.8 mL of the standard incubation, using a tetraphenylphosphonium (TPP^+^)-specific electrode as described previously [27]. The TPP^+^-electrode was calibrated with four sequential additions (0.4, 0.4, 0.8, and 1.6 µM) of TPP^+^.

To calculate the mΔΨ value, the matrix volume of yeast mitochondria was assumed to be 2 μL × mg^−1^ protein. The calculation assumes that TPP^+^ distribution between the mitochondria and the medium follows the Nernst equation. The mΔΨ values were corrected for TPP^+^ binding using the apparent external and internal partition coefficients of TPP^+^ [25]. This correction decreased the calculated mΔΨ values (approx. 30 mV shift), but it did not influence the changes in the resulting membrane potential (relative changes). Values of mΔΨ are given in mV.

As a respiratory substrate, 40 µM of NADH in the presence of an enzymatic regenerating system was used, i.e., 2 mM of glucose-6-phospate and 6 U of glucose-6-phosphate dehydrogenase. Phosphorylating respiration was measured using 150 μM of ADP (pulse). Only mitochondrial preparations with RCR of approximately 1.9–2.5 were used in the experiments. 

The proton leak measurements were performed under non-phosphorylating (resting state, state 4) conditions in the presence of 2 µM of CATR and 0.5 μg/mL of oligomycin, which inhibit the activities of the ADP/ATP antiporter and ATP synthase, respectively. To induce the AcUCP activity-mediated respiration, measurements were performed in the presence of 20 µM of linoleic acid. To inhibit AcUCP activity, GTP was added with a final concentration of 2 mM. 

### 4.5. Mitochondrial Transport of Succinate and Sulfate

Succinate transport by a dicarboxylate carrier was determined by measuring the rate of mitochondrial swelling in the presence of ammonium succinate. Mitochondria (0.2 mg) were re-suspended in 1 mL of iso-osmotic medium containing 0.125 M of ammonium succinate, 1 µM of antimycin A, and 10 mM of Tris/Cl at pH 6.8. Swelling was initiated by the addition of 5 mM of K_2_HPO_4_ [55].

Oxaloacetate carrier activity was determined by measuring the rate of mitochondrial swelling related to sulphate uptake. Oxaloacetate carrier is capable of transporting sulphate [56]. Mitochondria (0.2 mg) were re-suspended in 1 mL of iso-osmotic medium containing 0.125 M of ammonium sulfate, 1 µM of antimycin A, and 10 mM of Tris/Cl at pH 6.8.

The rate of mitochondrial swelling was measured spectrophotometrically at 540 nm using a UV 1620 Shimadzu spectrophotometer.

### 4.6. Viability of Yeast Cells under Oxidative Stress

Δ*SOD1* yeast cells (control and with pYES2 +AcUCP) were grown in 25 mL of the appropriate minimal selection medium (MSM-Ura containing 0.67% yeast nitrogen base, amino acids, 3% glycerol, and 2% D-galactose) without uracil up to the early exponential phase of growth (OD_550_ ~ 0.6–0.8) at 28 °C. Then, the cultures were treated with a final concentration of 10 mM of H_2_O_2_ and grown for 2 h. Cell viability was measured according to [75] by plating serial dilutions (undiluted, 10× diluted, 100× diluted, and 1000× diluted) of treated (by H_2_O_2_) and untreated yeast cells on MSM–Ura with a3% glycerol and 2% D-galactose and growing the cells at 28 °C or 37 °C for 3 days. After scan preparation, the density of yeast colonies was measured using the ImageJ, Version 1.54d, released 30 March 2023 (https://imagej.nih.gov, accessed on 30 June 2023) densitometry software.

### 4.7. Superoxide Anion Radical Level Measurement Using MitoSoxRed Fluorescent Dye

MitoSoxRed stock solution was prepared according to manufacturer’s instructions (ThermoFisher Scientific, Waltham, MA, USA): a 5 mM stock was prepared by dissolving the contents of the vial in 13 μL of anhydrous DMSO. The 1 μM working solution was prepared in a PBS buffer (Merck, Darmstadt, Germany). Yeast Δ*SOD1* (−AcUCP, +AcUCP) was cultured on the induction medium (MSM-Ura, 3% glycerol + 2% galactose) to the early logarithmic phase of growth (OD_600_ ~ 2–4); the corresponding volume with the 3 OD_600_ of each culture was centrifuged at 3000× *g* for 5 min, and the cell pellet was washed twice in sterile water, then re-suspended in 1 mL of PBS. The MitoSoxRed working solution was added and cells were incubated for 60 min at 37 °C to increase stress conditions. Afterwards, the cells were washed 2 times with sterile water. Fluorescence was determined using the Tekan SPARK plate reader with excitation/emission wavelengths of 510 nm/580 nm. The OD_600_ was measured at the same time using the Tekan SPARK plate reader.

### 4.8. Statistical Analysis

The results are expressed as the mean ± SEM obtained from at least three independent experiments, and each determination was performed at least in triplicate. One-Way ANOVA was used to identify any significant differences; differences were considered significant if *p* < 0.05 (*), *p* < 0.01 (**), or *p* < 0.001 (***).

### 4.9. Phylogenetic Analysis

The search for protein similarity was performed using BLAST (BLASTp) with the default search parameters [76] and non-redundant protein sequences database (all non-redundant GenBank CDS translations + PDB + SwissProt + PIR + PRF excluding environmental samples from WGS projects). Sequences compared were from *Amebozoa*: *Acanthamoeba castellanii* (AcUCP, XP_004338547.1), *Cavenderia fasciculata* (XP_004356144.1), *Dictyostelium discoideum* (XP_001733006.1); invertebrates: *Caenorhabditis elegans* (NP_505414.1), *Culex quinquefasciatus* (XP_001846101.1), *Daphnia magna* (KZS20933.1, XP_032790042.1), *Drosophila melanogaster* (NP_648501.1, NP_573246.1); vertebrates: *Bos taurus* (NP_777096.1, NP_001160000.1, NP_001039610.1), *Canis lupus familiaris* (NP_001003046.1, NP_001003048.1, NP_001271375.1), *Chelonia mydas* (EMP32084.1), *Danio rerio* (NP_571251.1, NP_956635.1, XP_017208245.1), *Gallus gallus* (NP_001012901.1), *Homo sapiens* (NP_001158889.1, NP_068605.1, NP_004268.3, AAC18822.1, NP_001269125.1, CAB59892.1), *Macaca mulata* (NP_001253948.1, NP_001182322.1, NP_001247778.1), *Mus musculus* (NP_033489.1, NP_033490.1, NP_038798.2, NP_077173.1, NP_035528.1, NP_082987.1, BAA32532.1), *Numida meleagris* (XP_021262336.1), *Ovis aries* (NP_001120745.1), *Pongo abelii* (NP_001124629.1), *Python bivittatus* (XP_007434620.1), *Rattus norvegicus* (NP_445953.1, NP_596909.1, NP_037299.1, NP_071793.2), *Xenopus tropicalis* (NP_001011241.1); plants: *Arabidopsis thaliana* (NP_190979.1, OAO96485.1), *Arabidopsis lyrata* (XP_020884108.1), *Brassica juncea* (ANQ91900.1), *Brassica rapa* (XP_009138231.1), *Capsella rubella* (XP_006285840.1), *Citrus clementina* (XP_006426287.1), *Cucumis sativus* (XP_004143448.1), *Cucumis melo* (XP_008448974.1), *Glycine soja* (KHN28792.1), *Glycine max* (XP_003516932.1, XP_003522752.1, XP_003519852.1), *Helianthus annuus* (XP_021987116.1), *Medicago truncatula* (XP_013460114.1), *Morus notabilis* (EXB89966.1, XP_024028813.1, XP_010104698.2), *Panicum miliaceum* (BAA08103.1), *Phaseolus vulgaris* (AHA84173.1), *Pistacia vera* (XP_031266159.1), *Rosa chinensis* (XP_024171301.1), *Saccharum officinarum* (AAU11471.1, AAU11462.1, AAU11465.1, AAU11466.1, AAU11463.1), *Solanum lycopersicum* (NP_001234756.2), *Theobroma cacao* (EOY33826.1), *Triticum aestivum* (BAB16385.1), *Zea mays* (NP_001182793.1); fungi: *Aspergillus flavus* (QRD84767.1), *Candida glabrata* (CEL56597.1), *Madurella mycetomatis* (KXX79971.1), *Neurospora crassa* (XP_956963.2), *Penicillium camemberti* (CRL22122.1), *Trichophyton rubrum* (OAL62317.1), *Yarrowia sp*. (KAG5366634.1), *Zymoseptoria brevis* (KJX97552.1). The multiple sequence alignments were achieved with the aid of ClustalW [77]. The evolutionary history was inferred by using the Maximum Likelihood method and the JTT matrix-based model [78]. Initial tree(s) for the heuristic search were obtained automatically by applying the Neighbour-Join and BioNJ algorithms to a matrix of pairwise distances estimated using the JTT model, and then selecting the topology with superior log likelihood value. Evolutionary analyses were conducted in MEGA11 [79].

## 5. Conclusions

The UCP of the amoeba *A. castellanii* (AcUCP) is the first and best functionally characterized UCP of unicellular eukaryotes. However, this is the first time that a link between the *Acucp* coding sequence and its protein product has been described. Our results suggest that AcUCP targeted to the yeast mitochondria may act as an antioxidant system through uncoupling activity. Phylogenetic analysis shows that the AcUCP diverged from other UCPs very early, but it is clearly localised within the UCP subfamily.

## Data Availability

Data are contained within the article or supplementary material.

## Supplementary Materials

The supporting information can be downloaded at: https://www.mdpi.com/article/10.3390/ijms241512501/s1.
