# Supplementary material for: Acanthamoeba castellanii Uncoupling Protein: A Complete Sequence, Activity, and Role in Response to Oxidative Stress
Source: Int J Mol Sci. 2023 Aug 6;24(15):12501. doi: 10.3390/ijms241512501 (PMC10419851; doi:10.3390/ijms241512501)
Supplement: Supplementary file 1 [file ijms-24-12501-s001.zip › ijms-2498231-supplementary.pdf]

Supporting Information

```
                           *        20         *        40         *        60         *        80         *       100
AcUCP          : -------------------------------ATGTCTGCAGGGAAGCGCACCCCCACGGAGACAAAACCGCTGCCTCTGTGGGCTGGGTGGCTTTGTGGT
NW_004457290   : ccaaccctatgcgcctgtcgcacagccggccatgtctgcagggaagcgcacccccacggagacaaaaccgctgcctctgtgggctgggtggctttgtggt
XM_004334150   : ----------------------------------------------------------------------------------------------------

                           *       120         *       140         *       160         *       180         *       200
AcUCP          : GGGCTGGCCGGATGCAACGCCGAGGCCTGGACCCTGCCCATCGACATCACCAAAGTCCGGCTTCAG----------------------------------
NW_004457290   : gggctggccggatgcaacgccgaggcctggaccctgcccatcgacatcaccaaagtccggcttcaggtagctttccatttcccacttgcccgtacccttc
XM_004334150   : ----------------------------------------------------------------------------------------------------

                           *       220         *       240         *       260         *       280         *       300
AcUCP          : ---------------------------------------------CTGCAAGGACAGCAGATGGCCCTGGCTGCCCAGGCCGGCGACTCCGTTGGTGCCG
NW_004457290   : gctactctgcgtgctgacttcgcacgtgtcgtgccgcttggttagctgcaaggacagcagatggccctggctgcccaggccggcgactccgttggtgccg
XM_004334150   : ------------------------------------------------------------atggccctggctgcccaggccggcgactccgttggtgccg

                           *       320         *       340         *       360         *       380         *       400
AcUCP          : TTCCGCTGGCTCCCAAGTACCGAGGGATGCTGCACGCCGGCGCCACCATTGTCCGCGAGGAAG-------------------------------------
NW_004457290   : ttccgctggctcccaagtaccgagggatgctgcacgccggcgccaccattgtccgcgaggaaggtataccttcactcagctgagagtggtgggcctggac
XM_004334150   : ttccgctggctcccaagtaccgagggatgctgcacgccggcgccaccattgtccgcgaggaag-------------------------------------

                           *       420         *       440         *       460         *       480         *       500
AcUCP          : -------------------------------GTGCGCTGAGCCTGTGGAAGGGCATCGCGCCGGCCCTGCTGCGGCAGTTCCTGTACACGGGCCTGCGCA
NW_004457290   : gcgtgcgtgcctcaacctcgcgcgcctgcaggtgcgctgagcctgtggaagggcatcgcgccggccctgctgcggcagttcctgtacacgggcctgcgca
XM_004334150   : -------------------------------gtgcgctgagcctgtggaagggcatcgcgccggccctgctgcggcagttcctgtacacgggcctgcgca

                           *       520         *       540         *       560         *       580         *       600
AcUCP          : TGGGAATCTACGAGCCCATCCGCAACTTCTTCGCCTTTGGCGGCACCAAGGCCTCCGACGCGCCACTGCTGACCAAGATCCTGGCCG-------------
NW_004457290   : tgggaatctacgagcccatccgcaacttcttcgcctttggcggcaccaaggcctccgacgcgccactgctgaccaagatcctggccggtaccgacaacta
XM_004334150   : tgggaatctacgagcccatccgcaacttcttcgcctttggcggcaccaaggcctccgacgcgccactgctgaccaagatcctggccg-------------

                           *       620         *       640         *       660         *       680         *       700
AcUCP          : --------------------------------------------------------------GAATGGTGGCCGGCGGAGTGTCGGCGGCGGTGTTCACC
NW_004457290   : cgagctactgtgtcgccactgtggcgcggtcataacgtgagcgtggcgtgatgctgcggcaggaatggtggccggcggagtgtcggcggcggtgttcacc
XM_004334150   : --------------------------------------------------------------gaatggtggccggcggagtgtcggcggcggtgttcacc

                           *       720         *       740         *       760         *       780         *       800
AcUCP          : CCGACGGACCTGCTGAAGGTGCGGATGCAGGGCTCCTCGGGCCAGCGGTACAGGAGCCTCCTGCACGCCATCAAGACCGTCGTGGCCGAGGAGAAGATCT
NW_004457290   : ccgacggacctgctgaaggtgcggatgcagggctcctcgggccagcggtacaggagcctcctgcacgccatcaagaccgtcgtggccgaggagaagatct
XM_004334150   : ccgacggacctgctgaaggtgcggatgcagggctcctcgggccagcggtacaggagcctcctgcacgccatcaagaccgtcgtggccgaggagaagatct

                           *       820         *       840         *       860         *       880         *       900
AcUCP          : CGGGCCTGTGGAAGGGCATGGGACCCACTTCGCAAAGAGCCGCAG-------------------------------------------------------
NW_004457290   : cgggcctgtggaagggcatgggacccacttcgcaaagagccgcaggtacccggccaacccaacccatcgctaccgtaccgactagctggtgtagattcac
XM_004334150   : cgggcctgtggaagggcatgggacccacttcgcaaagagccgcag-------------------------------------------------------

                           *       920         *       940         *       960         *       980         *      1000
AcUCP          : --------------------------------TTGTGGCTGCGGCCGAGCTGGCGACCTACGACCAGTGCAAGCAGTTCCTGCTGGGCAACAACATCATG
NW_004457290   : tgtaaatgttcatcgccgcctggatcctacagttgtggctgcggccgagctggcgacctacgaccagtgcaagcagttcctgctgggcaacaacatcatg
XM_004334150   : --------------------------------ttgtggctgcggccgagctggcgacctacgaccagtgcaagcagttcctgctgggcaacaacatcatg

                           *      1020         *      1040         *      1060         *      1080         *      1100
AcUCP          : CAGGACAACATCTACACCCACTTTGCCGCGTCCTTCATCGCGGGATTCGTGGCCACCGCCTCCTCGTCTCCCATCG------------------------
NW_004457290   : caggacaacatctacacccactttgccgcgtccttcatcgcgggattcgtggccaccgcctcctcgtctcccatcggtatgcctcgcgacctctcgtgca
XM_004334150   : caggacaacatctacacccactttgccgcgtccttcatcgcgggattcgtggccaccgcctcctcgtctcccatcggtatgcctcgcgacctctcgtgca

                           *      1120         *      1140         *      1160         *      1180         *      1200
AcUCP          : ---------------------------------------------------------------------ATGTGGTGAAGACGCGCGTGATGAATCAGCC
NW_004457290   : gcttccgtccgatcatcttcatcgttgatgctgatagcaccaaccggtccgatgtgcacattccaacagatgtggtgaagacgcgcgtgatgaatcagcc
XM_004334150   : gcttccgtccgatcatcttcatcgttgatgctgatagcaccaaccggtccgatgtgcacattccaacagatgtggtgaagacgcgcgtgatgaatcagcc

                           *      1220         *      1240         *      1260         *      1280         *      1300
AcUCP          : ATCGGACGCCAACGGCCGAGGGCTGTACTACCGTTCGTCGCTCGACTGCGCGCGCAAGCTGGTGGCCGCCGAGGGCGTGCGGGGCTTCTACCGTGGCTTC
NW_004457290   : atcggacgccaacggccgagggctgtactaccgttcgtcgctcgactgcgcgcgcaagctggtggccgccgagggcgtgcggggcttctaccgtggcttc
XM_004334150   : atcggacgccaacggccgagggctgtactaccgttcgtcgctcgactgcgcgcgcaagctggtggccgccgagggcgtgcggggcttctaccgtggcttc

                           *      1320         *      1340         *      1360         *      1380         *      1400
AcUCP          : CTGCCCAACTGGATTCGGTTGGGTCCCTGGAACATCATCATGTTCCTCACCTACGAGCAGCTACGACGGGTCGTCGAGAAGCACTAG-------------
NW_004457290   : ctgcccaactggattcggttgggtccctggaacatcatcatgttcctcacctacgagcagctacgacgggtcgtcgag-agcactagcgttcgttagcca
XM_004334150   : ctgcccaactggattcggttgggtccctggaacatcatcatgttcctcacctacgagcagctacgacgggtcgtcgag-agcactagcgttcgttag---


AcUCP          : --------
NW_004457290   : gcgcttcg
XM_004334150   : --------
```

Figure S1. Multiple alignment of *Acucp* complete coding sequence, sequence annotated as XM_004334150.1 and genomic DNA (NW_004457290). Matching genomic and cDNA sequences encoding AcUCP. The figure shows all five introns according to genomic sequence (NW_004457290: 13506..14912) highlighted in blue and one additional intron not yet annotated in XM_004334150.1 in red.

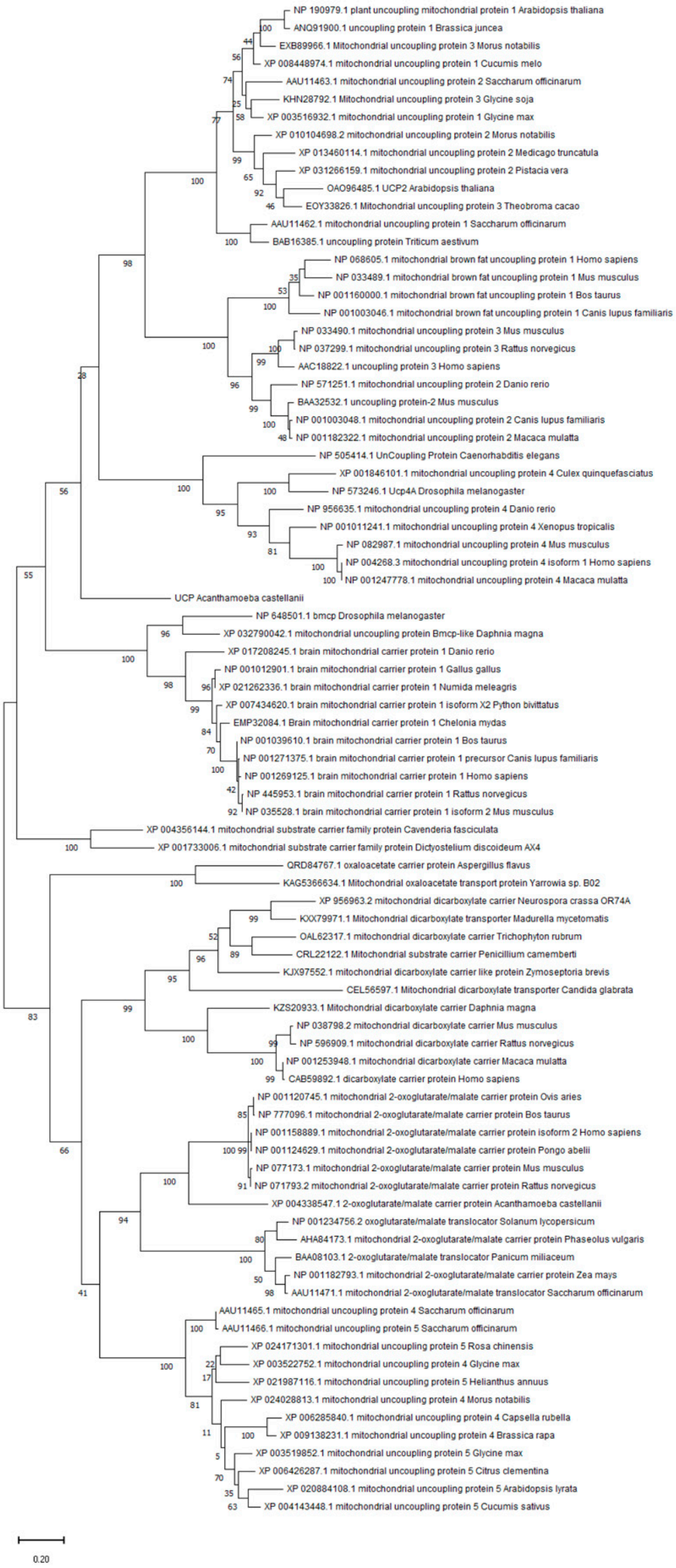

Figure S2. The phylogenetic relationships of MACPs with highest homology to UCPs.

The results were obtained by Maximum Likelihood method and JTT matrix-based model. The tree with the highest log likelihood (-24952.29) is shown. This analysis involved 85 amino acid sequences. There were 537 positions in the final dataset. Evolutionary analyses were conducted in MEGA11.

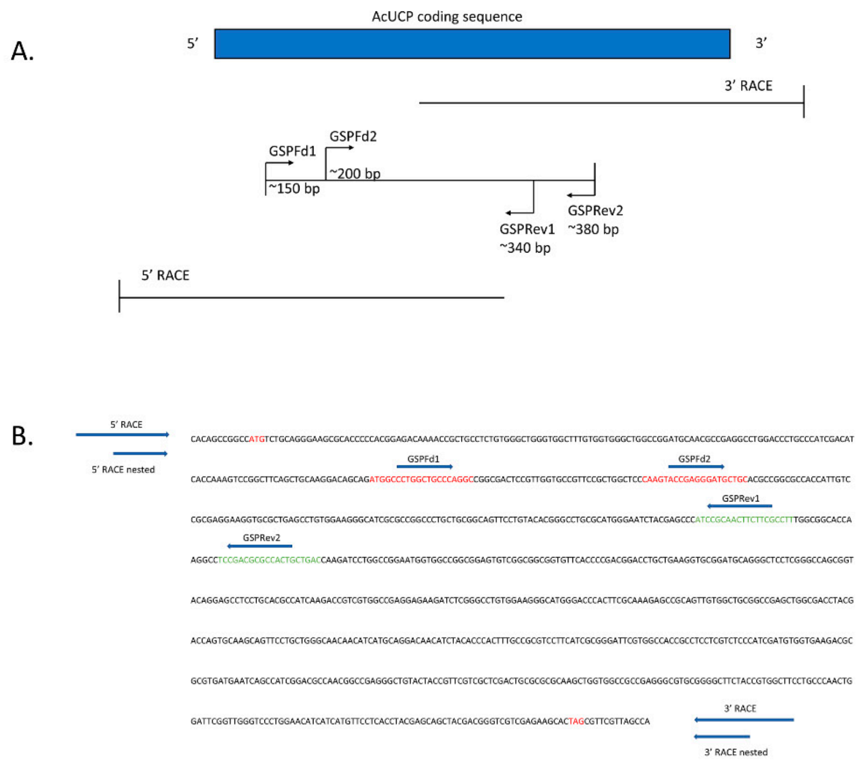

Figure S3 A. Schematic diagram of cloning strategy used to obtain *Acanthamoeba castellanii* uncoupling protein (AcUCP) coding sequence. RT-PCR using the 3' RACE and GSPFd1 primers provided an approximately 800 bp fragment confirmed by GSPFd2 and 3' RACE nested primers. The GSPRev1 and GSPRev2 primers allowed sequence extension using the 5' RACE primer and the nested 5' RACE primer in the same manner. It enabled design of primers for the entire sequence. (RACE, rapid amplification of cDNA ends.) **B. AcUCP coding sequence with the specific primers used in the RACE technique highlighted.** Sequencing of the resulting 3' RACE and 5′ RACE products enabled the assembly of the entire coding sequence.

**medium**	**glucose**				**glycerol**				**glycerol + galactose**			
**strain**	**InvSc1 Ctrl**	**InvSc1 + AcUCP**	**ΔSOD1 Ctrl**	**ΔSOD1 + AcUCP**	**InvSc1 Ctrl**	**InvSc1 + AcUCP**	**ΔSOD1 Ctrl**	**ΔSOD1 + AcUCP**	**InvSc1 Ctrl**	**InvSc1 + AcUCP**	**ΔSOD1 Ctrl**	**ΔSOD1 + AcUCP**
**doubling time [h]**	3.78 ± 0.05	3.70 ± 0.10	3.95 ± 0.05	3.88 ± 0.08	11.10 ± 0.40	9.50 ± 0.28	11.90 ± 0.37	10.1 ± 0.30	4.10 ± 0.06	4.40 ± 0.06	5.99 ± 0.08	4.50 ± 0.06***vs ΔSOD Ctrl

Table S1. Generation times of yeast cultures. Data are presented as mean ± SEM, ***** p<0,001**

	State 3	ΔΨm3	State 4	ΔΨm4	RCR
control	615 ± 34	193 ± 1.7	254 ± 14	221.4 ± 1	2.42 ± 0.18
+AcUCP	636 ± 39	190 ± 4.5	310 ± 19*	217.3 ± 0.9*	2.05 ± 0.14*

Table S2. Respiratory rates, ΔΨm values and coupling parameters in control (empty pYES2, Ctrl) and AcUCP-containing (pYES2+*Acucp*, +AcUCP) InvSc1 yeast mitochondria.
The respiratory rates (in nmol O_2 x min^{-1} x mg^{-1} protein) and ΔΨm values (in mV) of state 3 (phosphorylating respiration) and state 4 (non-phosphorylating respiration following state 3) as well as corresponding respiratory control ratios (RCR) are presented as ± SEM, using 40 μM NADH as a respiratory substrate and self-regenerating system. Significant differences versus values obtained for control mitochondria are shown, * **p<0,05**.

	+ 20 μM LA		+ 2 mM GTP	
	State 4	ΔΨm4	State 4	ΔΨm4
control	554 ± 32	209.6 ± 3.4	346 ± 19	209.6 ± 3.4
+AcUCP	527 ± 13	206.3 ± 3.5	329 ± 24	206.3 ± 3.5

Table S3. Respiratory rates, ΔΨm values in control (empty pYES2, Ctrl) and AcUCP-containing (pYES2+*Acucp*, +AcUCP) InvSc1 yeast mitochondria.
The respiratory rates (in nmol O_2 x min^{-1} x mg^{-1} protein) and ΔΨm values (in mV) of state 4 (non-phosphorylating respiration using 40 μM NADH as a respiratory substrate and self-regenerating system, in the presence of oligomycin and CATR) after the addition of 20 μM LA followed by 2 mM GTP ± SEM are presented.

Supplementary materials: cloning to pYES2

The cloning of complete AcUCP protein coding sequence with N-His tag and C-His tag or without any tags into pYES2 yeast expressing vector with Vazyme ClonExpress II One Step Cloning system.

The Vazyme ClonExpress II One Step Cloning system (Vazyme Biotech Co.) enables directional insertion of any amplified DNA product into any linearized vector at any site, provided that there are overlapped sequences of 15 bp - 20 bp on both 5′- and 3′- ends.

Experimental procedure consists of the following steps:

1. PCR for 954-bp *Acucp* CDS amplicon was performed using Q5 Hot Start High Fidelity kit (New England Biolabs) according to manufacturer's instructions with primers AcUCP fd: 5′ATGTCTGCAGGGAAGCGCA3′ and AcUCP rev: 5′CTAGTGCTTCTCGACGACCC3′. The applied thermal cycling conditions consisted of an initial denaturation at 98°C for 1 min followed by 35 cycles at 98°C for 10 s, 68°C for 15 s, 72°C for 20 s, and ending incubation at 72˚C for 2 min. The PCR product was purified using QIAquick PCR Purification Kit (Qiagen) per the manufacturer's instructions.
2. Overhang PCRs
    a. Overhang PCR for adding of vector sequnces to *Acucp* CDS amplicon to produce UCPexp amplicon was performed using Q5 Hot Start High Fidelity kit (New England Biolabs) according to manufacturer's instructions.
    Primer pair used for UCPexp amplicon: FducpexpYES2 (5'-TTGGTACCGAGCTCGGATCCATGTCTGCAGGGAAGCGCA-3') and RevucpexpYES2 (5'-GATGGATATCTGCAGAATTCCTAGTGCTTCTCGACGACCC-3'). The applied thermal cycling conditions consisted of an initial denaturation at 98 °C for 2 min followed by 35 cycles at 98°C for 10 s, 72°C for 45 s and ending incubation at 72˚C for 2 min. The PCR product was purified using QIAquick PCR Purification Kit (Qiagen) according to the manufacturer's instructions.

b. Overhang PCR1 for adding of His-tag, Gly linker and vector sequences to *Acucp* CDS amplicon to produce UCP His-N/C amplicons was performed using Q5 Hot Start High Fidelity kit (New England Biolabs) according to manufacturer's instructions.

    i. For amplification of sequence encoding AcUCP tagged with six histidine residues at the N-terminus to produce UCP_His-N amplicon was used primer pair: AcucpHF (5' GAGCTCGGATCCATGCATCATCACCATCACCACGGAGGTGGAATGTCTGCAGG GAAGCGCA 3') and Acucpecr (5' CCGGAATTCCTAGTGCTTCTCGACGACCC 3'). The applied thermal cycling conditions consisted of an initial denaturation at 98°C for 1 min followed by 5 cycles at 98°C for 10 s, 72°C for 15 s, 72°C for 35 s, then 30 cycles at 98°C for 10 s, 70°C for 15 s, 72°C for 35 s, and ending incubation at 72˚C for 2 min. The PCR product was purified using QIAquick PCR Purification Kit (Qiagen) as per the manufacturer’s instructions.

    ii. For amplification of sequence encoding AcUCP tagged with six histidine residues at the C-terminus to produce UCP_His-C amplicon primer pair used was: Acucpbaf (5' GCGGGATCCATGTCTGCAGGGAAGCGCA 3') and AcucpHR (5' CTGCAGAATTCTTAGTGGTGATGGTGATGATGACCTCCACCGTGCTTCTCGACG ACCCGT 3'). The applied thermal cycling conditions consisted of an initial denaturation at 98°C for 1 min followed by 5 cycles at 98°C for 10 s, 72°C for 15 s, 72°C for 35 s, then 30 cycles at 98°C for 10 s, 70°C for 15 s, 72°C for 35 s, and ending incubation at 72˚C for 2 min. The PCR product was purified using QIAquick PCR Purification Kit (Qiagen) as per the manufacturer’s instructions.

c. Overhang PCR2 for extension of vector sequences in UCP_His-N or UCP_His-C amplicon to produce UCP_His-N2/C2 amplicons was performed using Q5 Hot Start High Fidelity kit (New England Biolabs) according to manufacturer's instructions.

    i. For UCP_His-N2 amplicon, primer pair used: AcucpHF2 (5' CTTGGTACCGAGCTCGGATCCATGCATCA 3') and Acucpecr2 (5' TGATGGATATCTGCAGAATTCCTAGTGCTTCTCGACGA 3'). The applied thermal cycling conditions consisted of an initial denaturation at 98°C for 1 min followed by 5 cycles at 98°C for 10 s, 67°C for 15 s, 72°C for 35 s, then 30 cycles at 98°C for 10 s, 72°C for 35s, and ending incubation at 72˚C for 2 min. The band of PCR product excised from agarose was purified using the Monarch DNA Gel Extraction Kit (New England BioLabs) and QIAquick PCR Purification Kit (Qiagen) per the manufacturer’s instructions.

    ii. For UCP_His-N2 amplicon, primer pair used: Acucbaf2 (5' CTTGGTACCGAGCTCGGATCCATGTCTGCAGGGAA 3') and AcucpHR2 (5' TGATGGATATCTGCAGAATTCTTAGTGGTGATGG 3'). The applied thermal cycling conditions consisted of an initial denaturation at 98°C for 1 min followed by 5 cycles at 98°C for 10 s, 66°C for 15 s, 72°C for 35 s, then 30 cycles at 98°C for 10 s, 70°C for 15 s, 72°C for 35 s, and ending incubation at 72˚C for 2 min. The excised from agarose band of PCR product was purified using the Monarch DNA Gel Extraction Kit (New England BioLabs) and QIAquick PCR Purification Kit (Qiagen) according to the manufacturer’s instructions.

3. The pYES2 vector (ThermoFisher Scientific) was prepared for cloning by its digestion with restriction enzymes at the cloning site. Restriction of pYES plasmid with BamHI-HF (New England Biolabs) and EcoRI-HF (New England Biolabs) was carried out according to restriction enzyme manufacturer's instructions. Purification of digested plasmid (vector backbone) was carried out using QIAquick PCR Purification Kit (Qiagen) according to manufacturer's instructions.

4. The cloning was performed using ClonExpress II One Step Cloning kit (Vazyme Biotech Co.) according to manufacturer's instructions. The purified preparations of the linearized vector and UCPexp, UCP_His-N2 or UCP_His-C2 amplicon were used for recombination to yield pYES2+*Acucp*, pYES2+*N-HisAcucp*, pYES2+*C-HisAcucp* vector, respectively.

5. Transformations of competent DH5α *E.coli* cells with recombinant vectors were performed using standard procedure.

6. *Colony PCR to screen for* positive transformants was carried out on *ampicillin-selected clones* lysed by boiling. Reaction was performed using 5x HOT FIREPol Blend Master Mix (Solis BioDyne OÜ) and vector-specific primer pair: YESfor (5' CGGATCGGACTACTAGCAG 3') and Cyc1rev (5' GTGAATGTAAGCGTGACATAAC 3'). The applied thermal cycling conditions consisted of an initial denaturation at 98°C for 12 min followed by 35 cycles at 98°C for 10 s, 54°C for 15 s, 72°C for 75 s, and ending incubation at 72˚C for 5 min.

7. Isolations of successfully recombinant plasmids was carried out using 12 mL overnight *E. coli* DH5alpha liquid cultures and the Pasmid Mini AX kit (A@A Biotechnology) according to manufacturer's instructions.

8. DNA sequencing of some chosen colony PCR products and then of isolated plasmid were performed to validate target recombinants.

9. DNA sequencing was undertaken using the ABI Prism Big Dye Terminator Sequencing Kit version 3.1 (Applied Biosystems, Foster City, CA, USA). Cycle sequencing was performed in 20 µl reaction volume containing appropriate amount of DNA template and 1.5 pmole of primer, using the kit supplier's recommended thermal profile in a 2720 Thermal Cycler. After purification of the extension products by ethanol/EDTA precipitation method the DNA sequence was read by capillary electrophoresis performed in an ABI Prism 3130XL Genetic Analyser (Applied Biosystems, Foster City, CA, USA).

10. Sequence Scanner software v2.0 (Applied Biosystems) was used to check chromatograms and to control the data quality. Assembly of the sequence reads to generate consensus sequence and to compare it to a known reference sequence was performed using CodonCode Aligner software v10.0.2 (CodonCode Corporation).
